# Supplementary material for: Single-cell RNA sequencing reveals hemocyte heterogeneity, differentiation trajectories, and viral tropism in shrimp (Macrobrachium rosenbergii) infected with decapodiridovirus litopenaeus1
Source: J Virol. 2025 Jul 18;99(8):e00790-25. doi: 10.1128/jvi.00790-25 (PMC12363182; doi:10.1128/jvi.00790-25)
Supplement: Supplemental figures — Figures S1 to S8. [file jvi.00790-25-s0001.docx]

**Supplementary Figures and Figure Legends**

**Supplementary Figure 1**

**
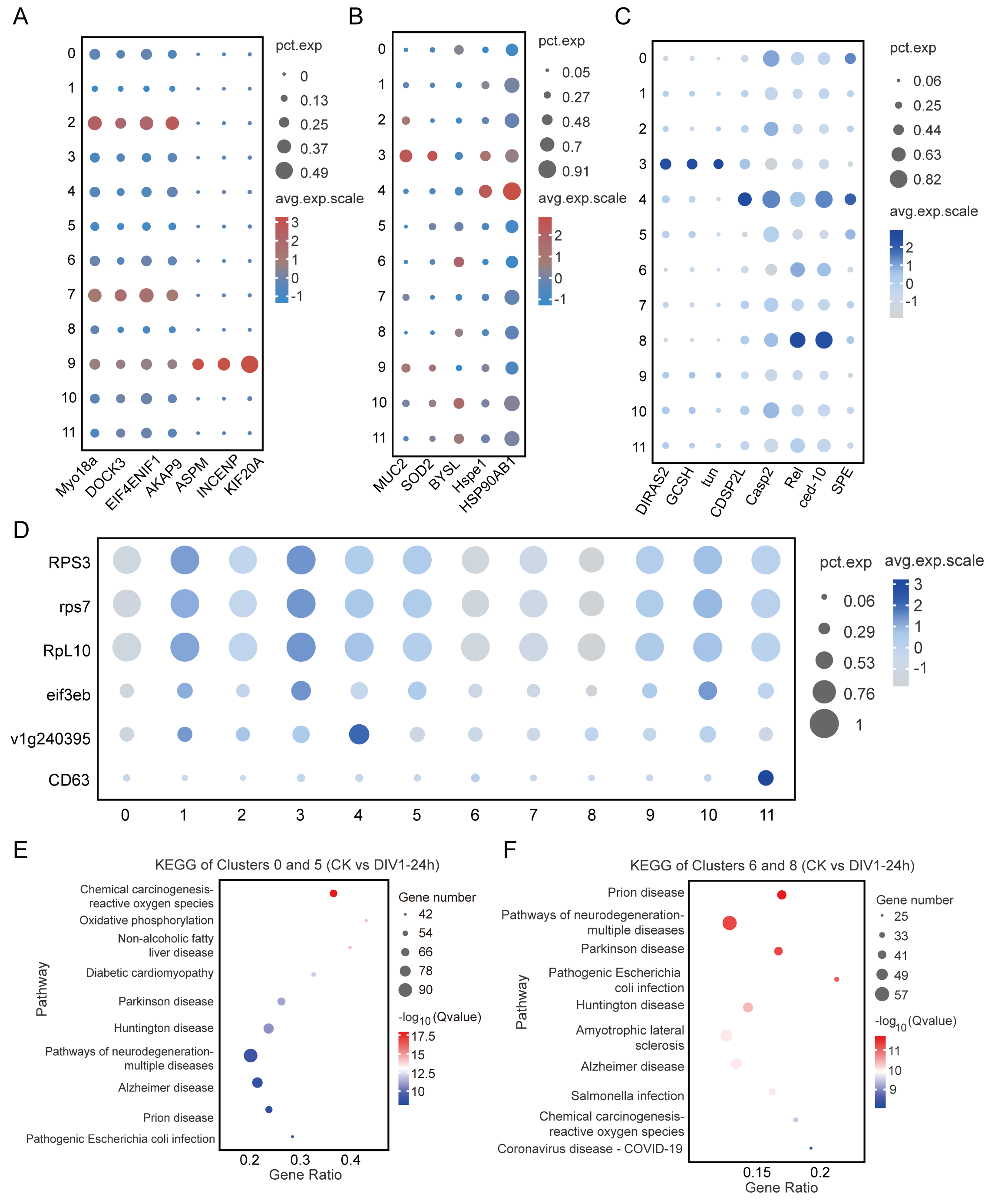
**

**Supplementary Figure 1. Genes especially high expressed in clusters 1, 2, 3, 4, 7, 9, 10, 11 and KEGG analysis of DEGs between group CK and DIV1-24h in clusters 0, 5 and 6, 8.**

(A) The high expression distribution of genes related to cell proliferation, division, and germline development in clusters 2, 7, and 9.

(B) DEGs related to cytoskeletal formation, immune stress and cell development in cluster 3 ,4 and 10.

(C) DEGs with SGC characterization in cluster 3, 4.

(D) Expression profiles of key DEGs related to protein synthesis (*Rps3, rps7, RpL10, eif3eb, v1g240395*) and phagocytosis (*CD63*).

(E, F) TOP 10 KEGG pathway enrichment analysis of differential genes in clusters 0 and 5 (E), clusters 6 and 8 (F) compared to the control group at 24 hpi. Dot size represents the number of genes involved, and color intensity indicates statistical significance.

**Supplementary Figure 2**

**
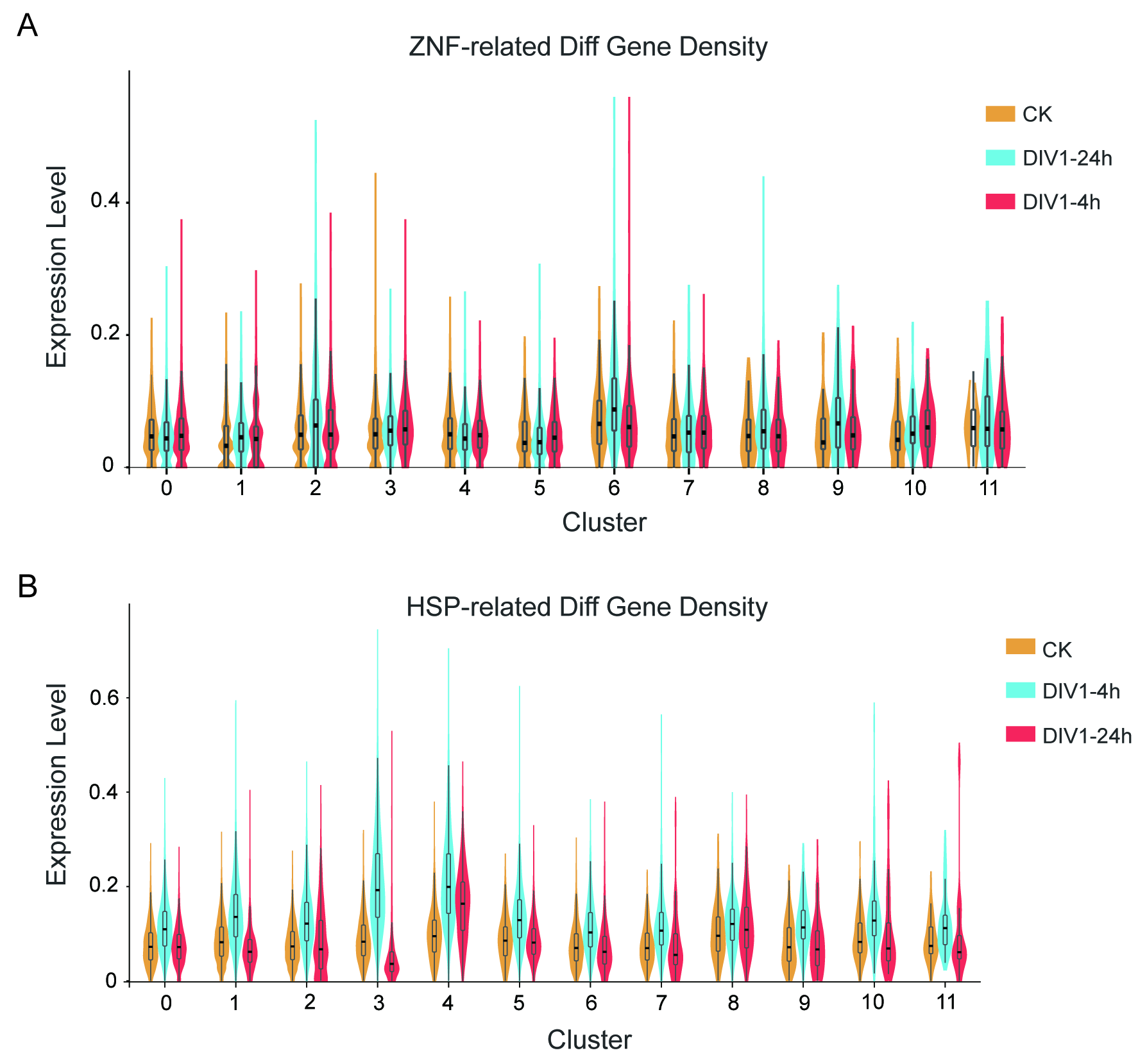
**

**Supplementary Figure 2. Expression profile of DEGs related to heat stress response or gene regulation between control group and DIV1-infection groups in each cluster.**

(A) The distribution of differentially expressed zinc finger proteins (ZNFs) in each cluster in different groups.

(B) The distribution of differentially expressed heat shock proteins (HSPs) in each cluster in different groups.

**Supplementary Figure 3**

**
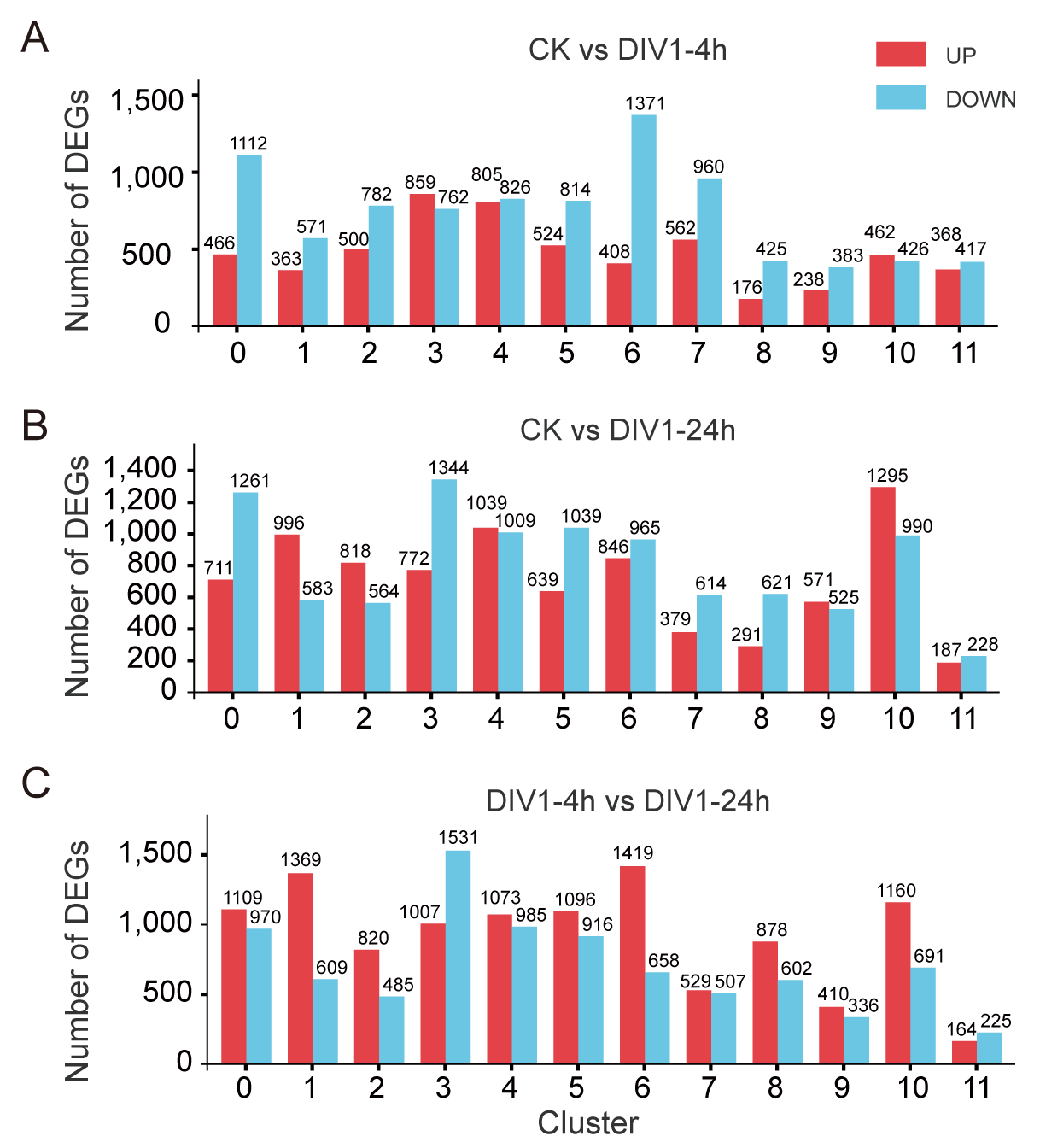
**

**Supplementary Figure 3. Overview of DEGs between DIV1-infected and control cells within shrimp hemocyte clusters.**

(A, B) Histogram showing all of the upregulated (red) and downregulated DEGs (blue) in DIV1-infected cells compared with control cells within all clusters at 4 (A) and 24 hpi (B).

1. Histogram showing all of the upregulated (red) and downregulated DEGs (blue) in group DIV1-24h cells compared with DIV1-4h cells.

**Supplementary Figure 4**

**
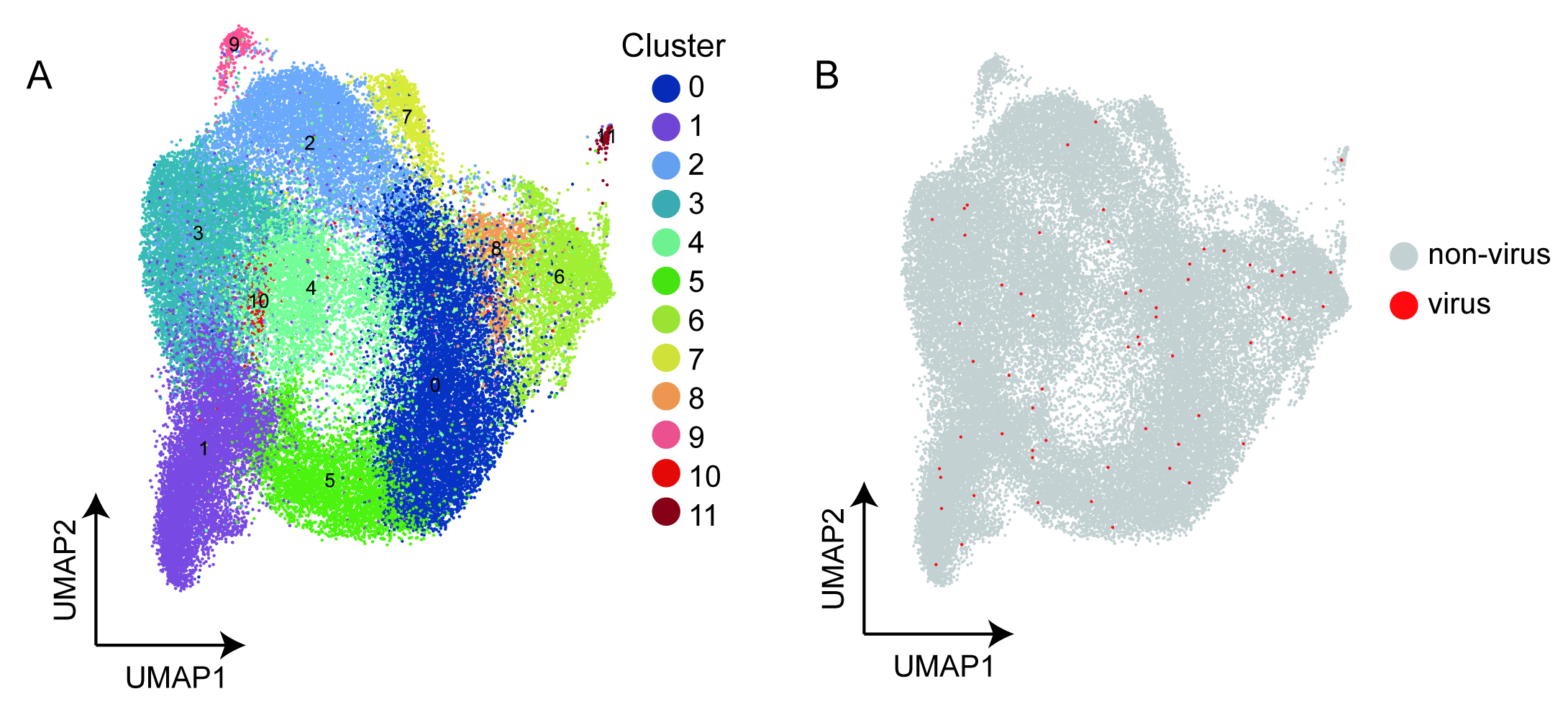
**

**Supplementary Figure 4. Detection of DIV1 genes of hemocyte cells within all clusters in DIV1-infected groups.**

1. UMAP projection representing the 12 cell clusters identified in the shrimp hemocytes (unified set of control and DIV1 infection samples).
2. UMAP plot showing the expression atlas of all cells detected with virus genes in DIV1-infected samples, the dots in red represent the infected cells.

**Supplementary Figure 5**

**
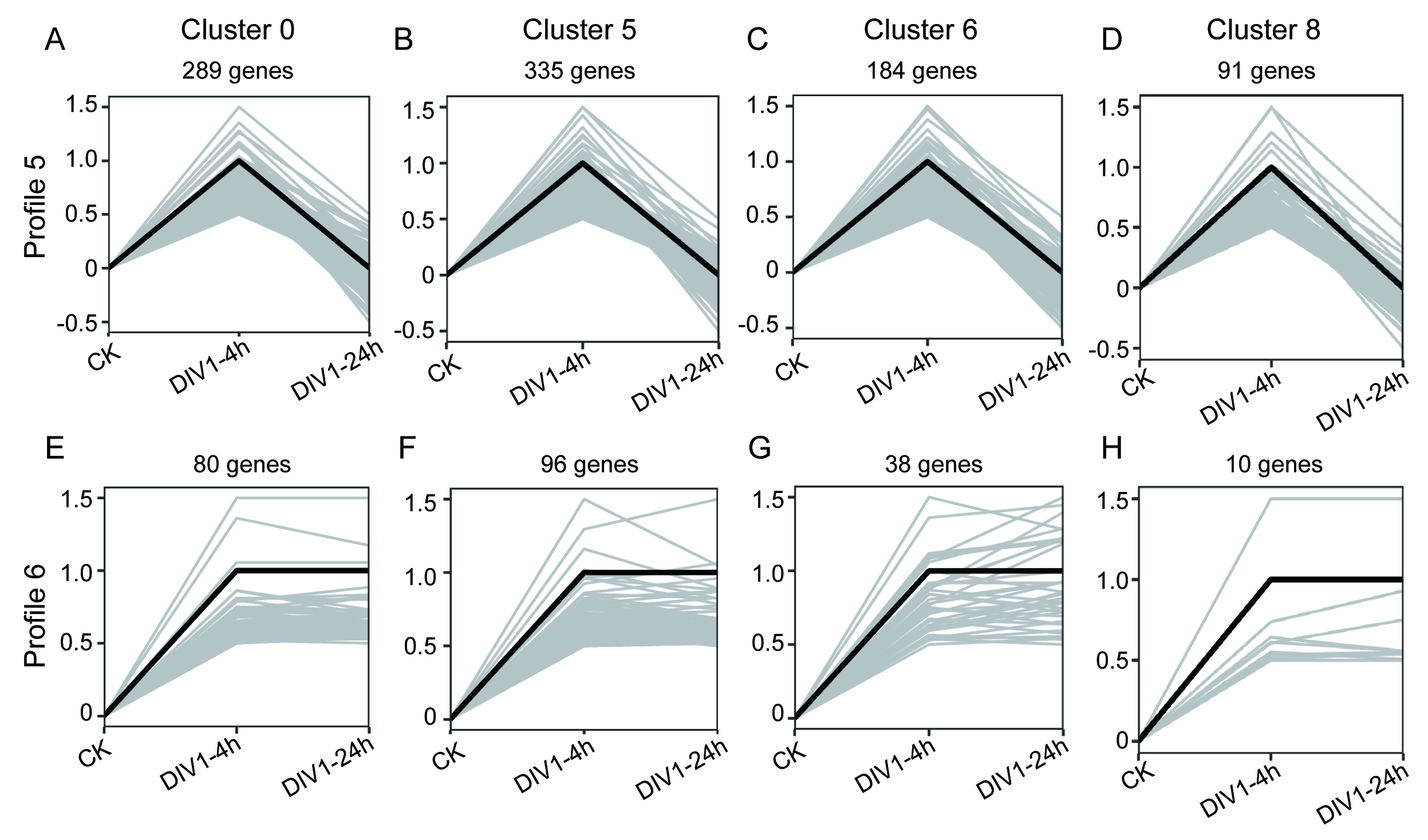
**

**Supplementary Figure 5. Patterns of gene expressions across three groups of hemocytes inferred by STEM analysis**.

(A-D) Profile 5 represented genes, in cluster 0 (A), cluster 5 (B), cluster 6 (C) and cluster 8 (D), which were highly up-regulated in group DIV1-4h. In each profile, the light gray lines represented the expression pattern of each gene, while the black line represented the expression tendency of all the genes. The number of genes belonging to each pattern was labeled above the profile.

(E-H) Profile 6 represented genes, in cluster 0 (E), cluster 5 (F), cluster 6 (G) and cluster 8 (H), respectively, that were highly up-regulated in both groups DIV1-4h and DIV1-24h.

**Supplementary Figure 6**

**
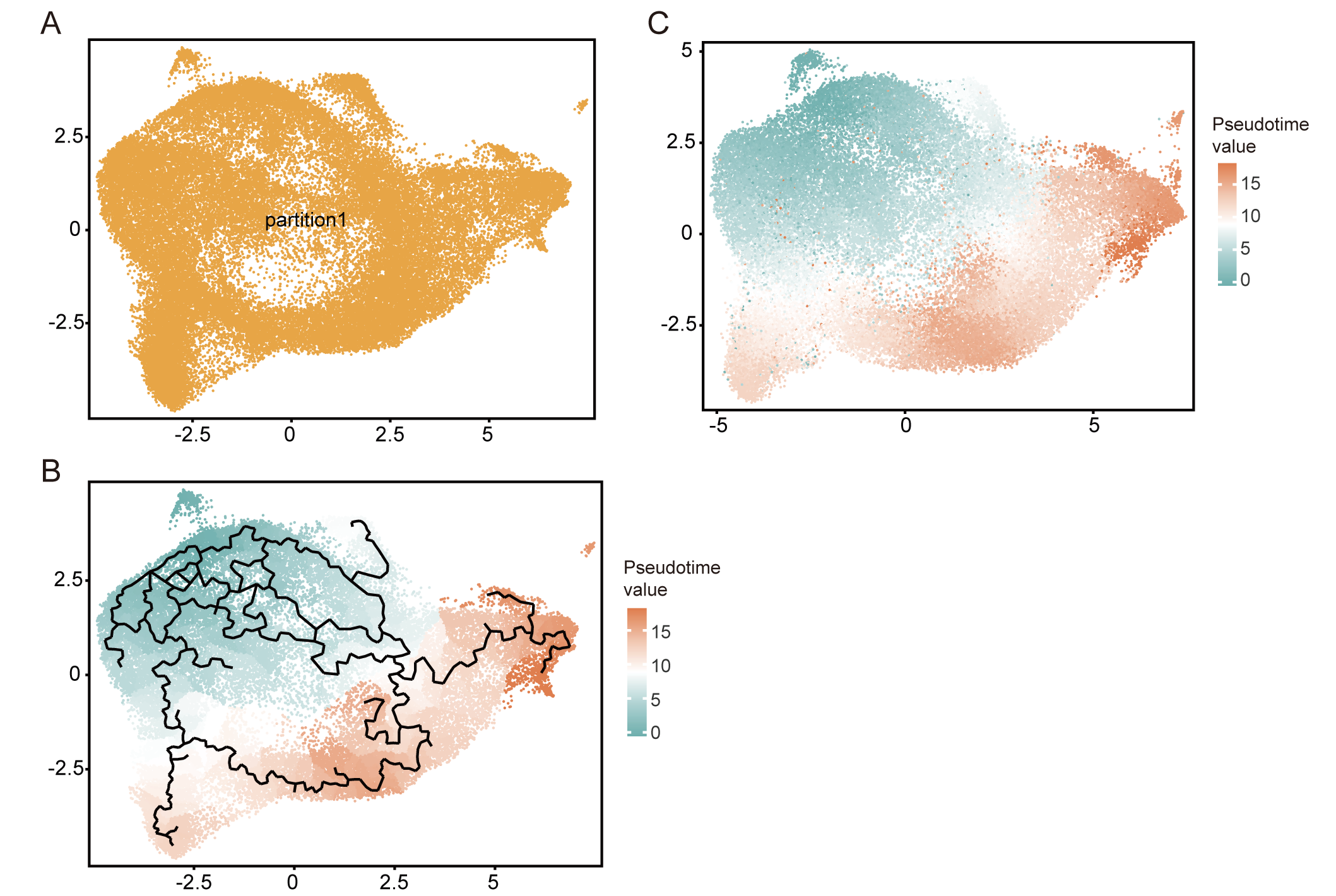
**

**Supplementary Figure 6. Pseudotime trajectory of hemocytes in shrimp *M. rosenbergii* by Monocle 3 analysis.**

(A) The profile of differentiation partition in the single cell atlas.

(B) The differentiation trajectory among different clusters mapped in single cell atlas. Black lines indicate differentiation trajectories, and the higher the pseudotime value, the darker the color. Each point represents a single cell.

(C) The mapping of pseudotime values in single cell atlas. The higher the pseudotime value, the greater the degree of differentiation. Each point represents a single cell.

**Supplementary Figure 7**

**
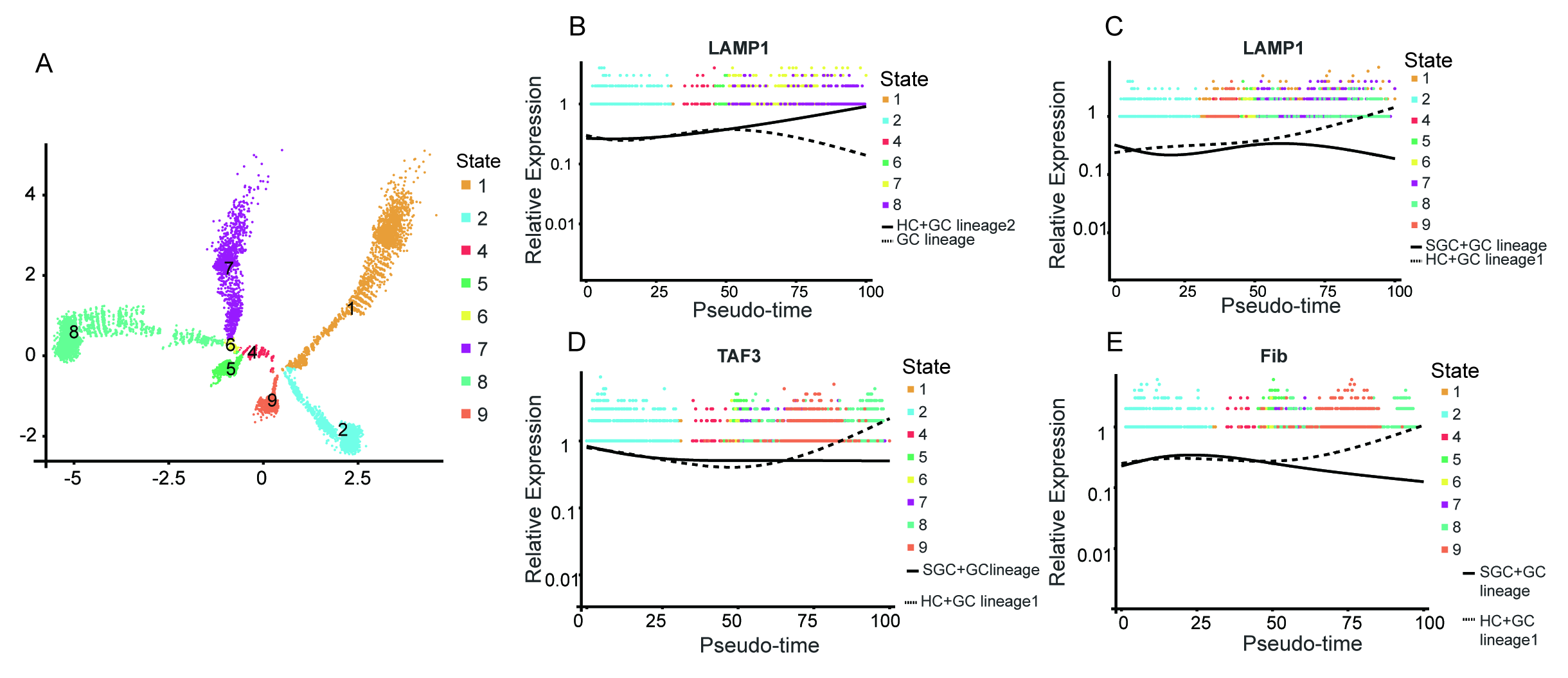
**

**Supplementary Figure 7. Trajectory distribution of genes of *LAMP1*, *TAF3*, and *Fib*.**

(A) Trajectory distribution of different differentiation states.

(B, C) The expression trend of gene *LAMP1* in differentiation to HC branch 2 (B) or branch 1 (C) and other branches.

(D, E) The expression trend of transcription factor *TAF3* (D)*、Fib* (E) in differentiation to HC branch and other branches.

**Supplementary Figure 8**

**
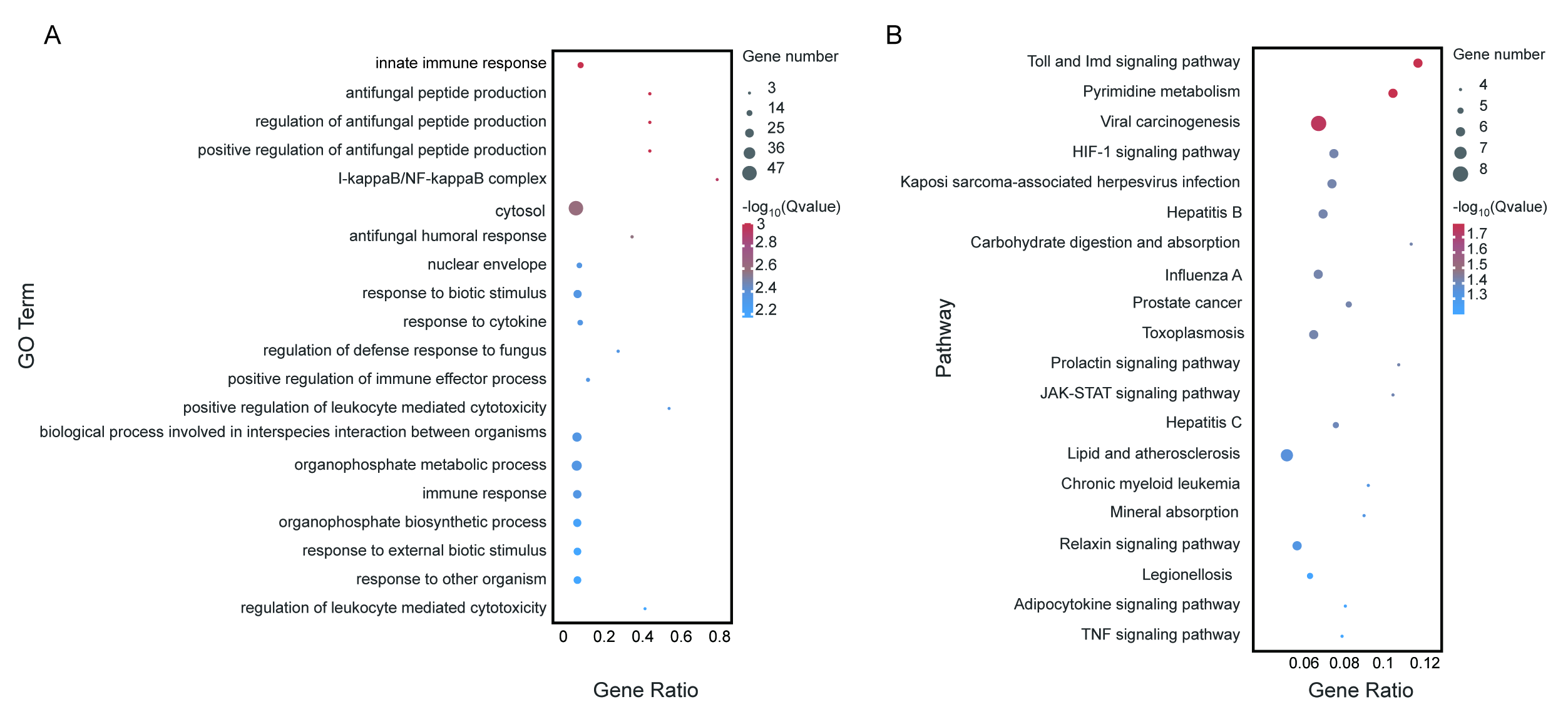
**

**Supplementary Figure 8. Function annotation of differential fate genes in branch 2 after HC re-clustering.**

(A, B) GO (A) and KEGG (B) enrichment analysis of differential fate genes in branch 2 after regrouping of hyalinocyte clusters.
